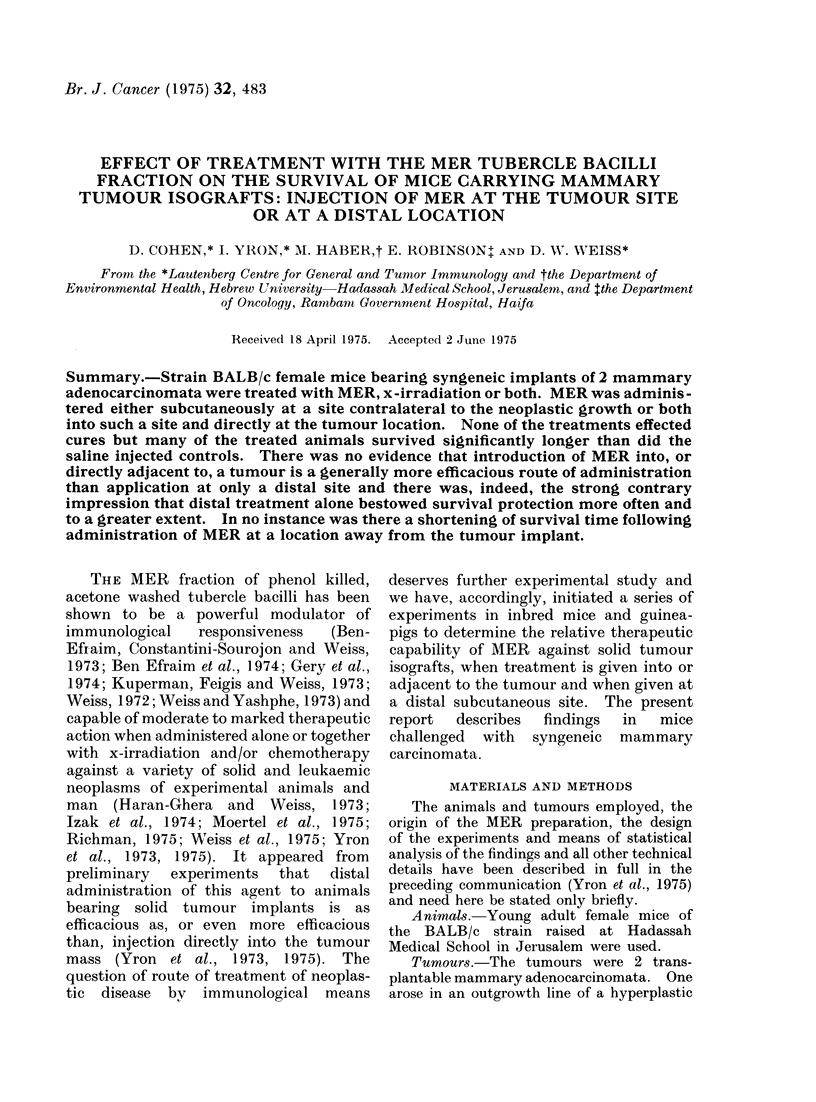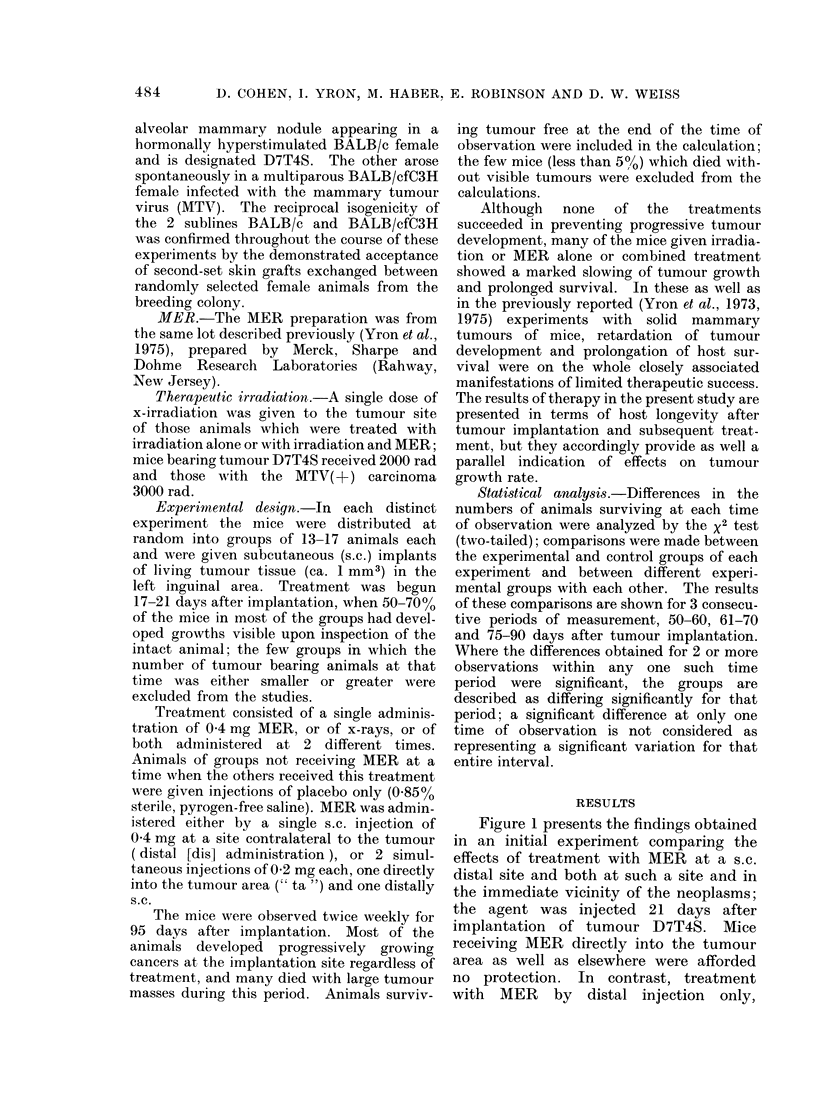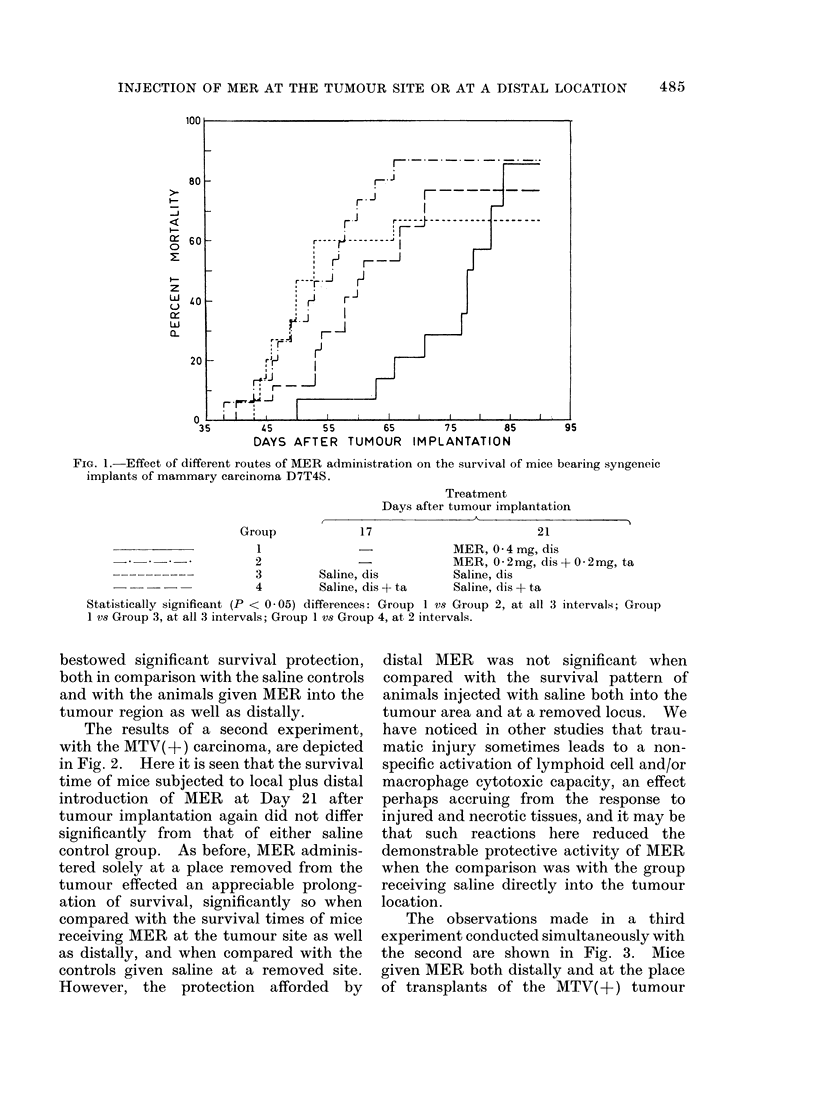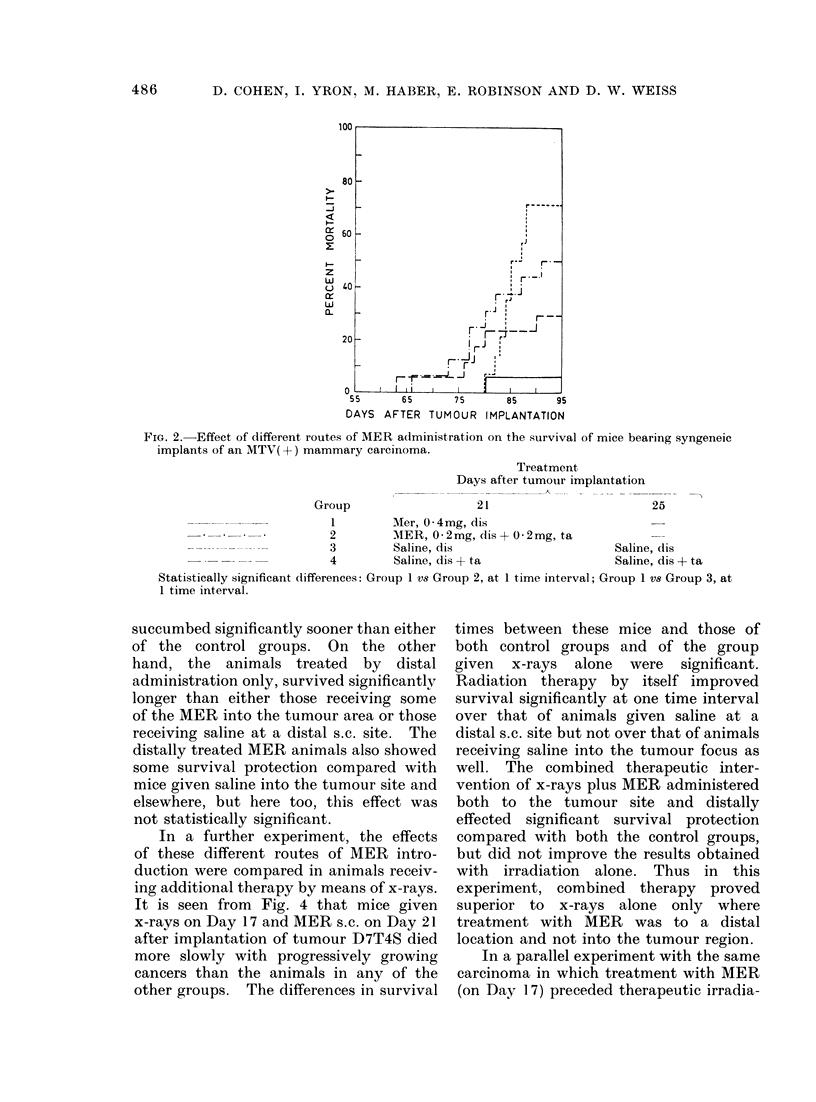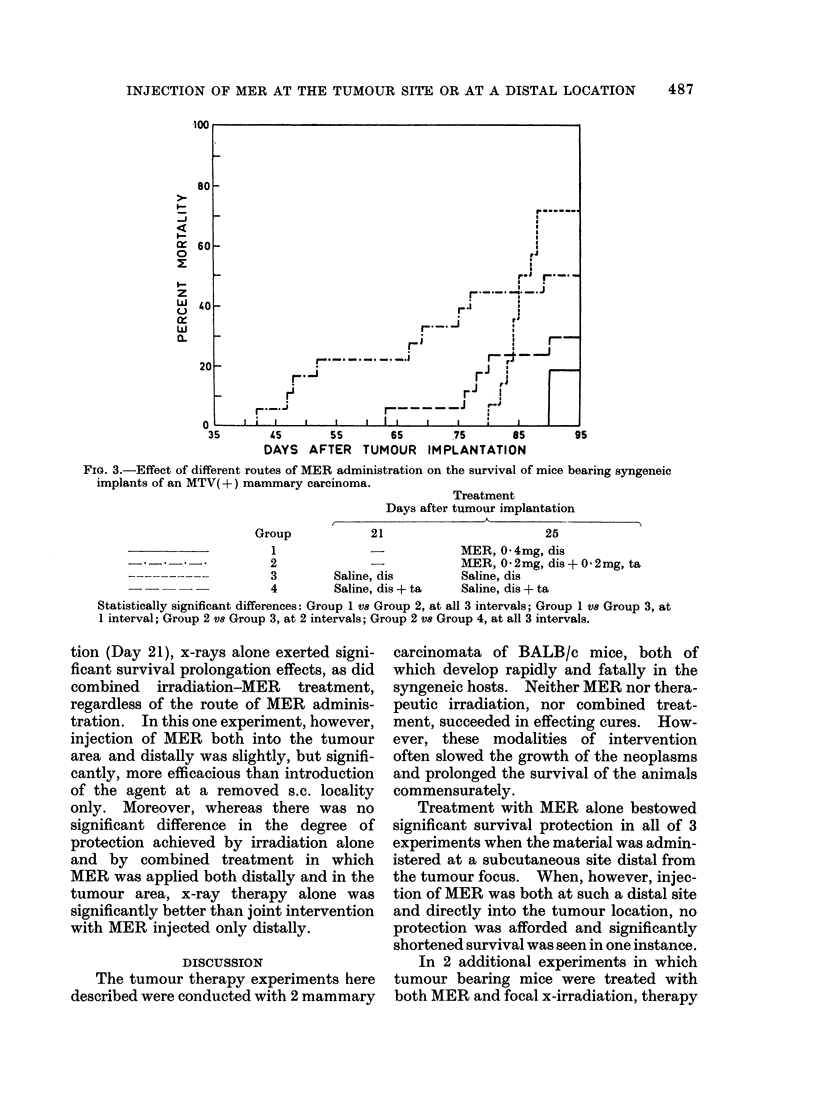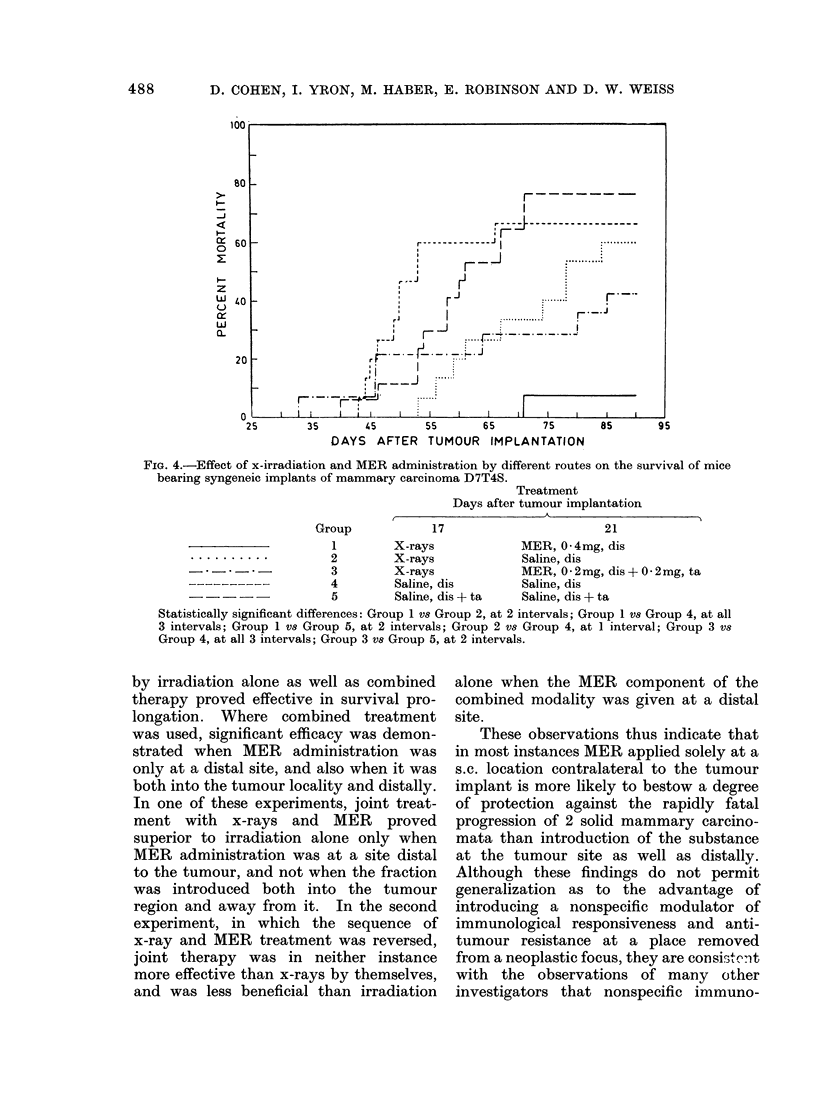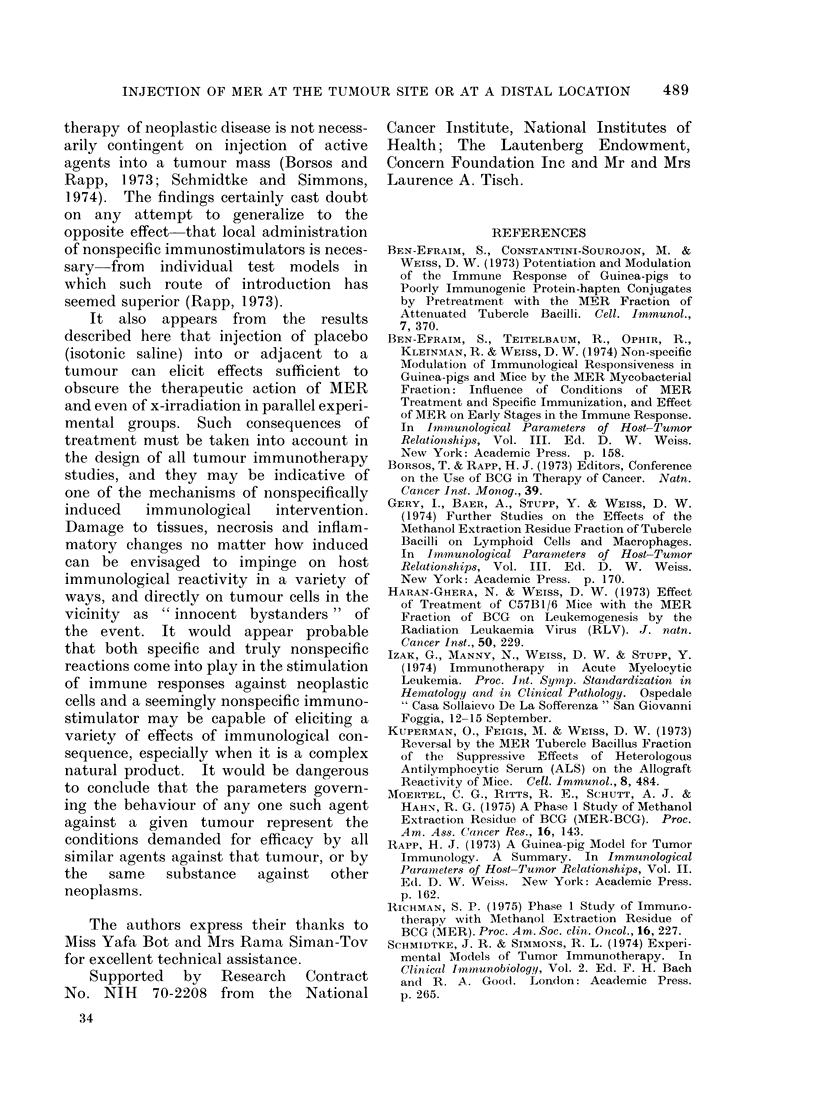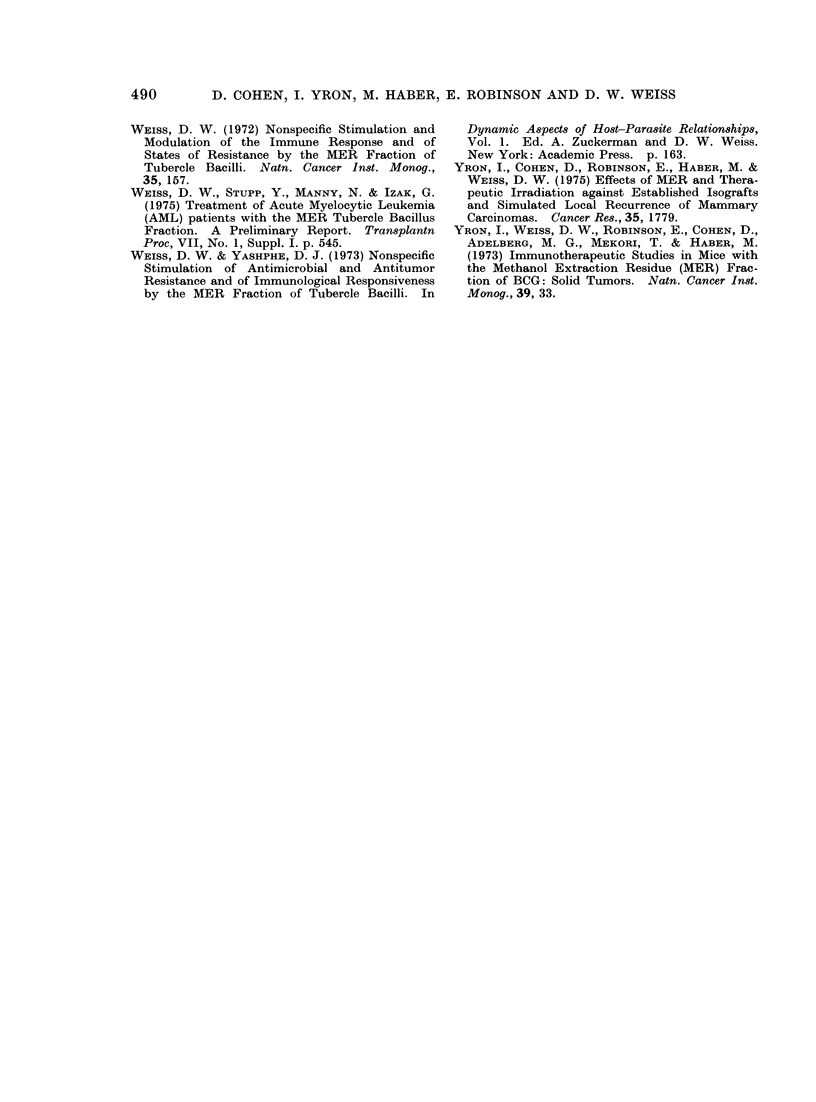# Effect of treatment with the MER tubercle bacilli fraction on the survival of mice carrying mammary tumour isografts: injections of MER at the tumour site or at a distal location.

**DOI:** 10.1038/bjc.1975.250

**Published:** 1975-10

**Authors:** D. Cohen, L. Yron, M. Haber, E. Robinson, D. W. Weiss

## Abstract

Strain BALB/c female mice bearing syngeneic implants of 2 mammary adenocarcinomata were treated with MER, x-irradiation or both. MER was administered either subcutaneously at a site contralateral to the neoplastic growth or both into such a site and directly at the tumour location. None of the treatments effected cures but many of the treated animals survived significantly longer than did the saline injected controls. There was no evidence that introduction of MER into, or directly adjacent to, a tumour is a generally more efficacious route of administration than application at only a distal site and there was, indeed, the strong contrary impression that distal treatment alone bestowed survival protection more often and to a greater extent. In no instance was there a shortening of survival time following administration of MER at a location away from the tumour implant.


*Br. J. Cancer* (1975) **32**, 483

### EFFECT OF TREATMENT WITH THE MER TUBERCLE BACILLI FRACTION ON THE SURVIVAL OF MICE CARRYING MAMMARY TUMOUR ISOGRAFTS: INJECTION OF MER AT THE TUMOUR SITE OR AT A DISTAL LOCATION

D. COHEN,* I. YRON,* M. HABER,† E. ROBINSON‡ AND D. W. WEISS*

*From the *Lautenberg Centre for General and Tumor Immunology and †the Department of Environmental Health, Hebrew University—Hadassah Medical School, Jerusalem, and ‡the Department of Oncology, Rambam Government Hospital, Haifa*

Received 18 April 1975. Accepted 2 June 1975

**Summary.—Strain BALB/c female mice bearing syngeneic implants of 2 mammary adenocarcinomata were treated with MER, x-irradiation or both. MER was administered either subcutaneously at a site contralateral to the neoplastic growth or both into such a site and directly at the tumour location. None of the treatments effected cures but many of the treated animals survived significantly longer than did the saline injected controls. There was no evidence that introduction of MER into, or directly adjacent to, a tumour is a generally more efficacious route of administration than application at only a distal site and there was, indeed, the strong contrary impression that distal treatment alone bestowed survival protection more often and to a greater extent. In no instance was there a shortening of survival time following administration of MER at a location away from the tumour implant.**

THE MER fraction of phenol killed, acetone washed tubercle bacilli has been shown to be a powerful modulator of immunological responsiveness (Ben-Efraim, Constantini-Sourojon and Weiss, 1973; Ben Efraim *et al.*, 1974; Gery *et al.*, 1974; Kuperman, Feigis and Weiss, 1973; Weiss, 1972; Weiss and Yashphe, 1973) and capable of moderate to marked therapeutic action when administered alone or together with x-irradiation and/or chemotherapy against a variety of solid and leukaemic neoplasms of experimental animals and man (Haran-Ghera and Weiss, 1973; Izak *et al.*, 1974; Moertel *et al.*, 1975; Richman, 1975; Weiss *et al.*, 1975; Yron *et al.*, 1973, 1975). It appeared from preliminary experiments that distal administration of this agent to animals bearing solid tumour implants is as efficacious as, or even more efficacious than, injection directly into the tumour mass (Yron *et al.*, 1973, 1975). The question of route of treatment of neoplastic disease by immunological means deserves further experimental study and we have, accordingly, initiated a series of experiments in inbred mice and guinea-pigs to determine the relative therapeutic capability of MER against solid tumour isografts, when treatment is given into or adjacent to the tumour and when given at a distal subcutaneous site. The present report describes findings in mice challenged with syngeneic mammary carcinomata.

### MATERIALS AND METHODS

The animals and tumours employed, the origin of the MER preparation, the design of the experiments and means of statistical analysis of the findings and all other technical details have been described in full in the preceding communication (Yron *et al.*, 1975) and need here be stated only briefly.

*Animals.*—Young adult female mice of the BALB/c strain raised at Hadassah Medical School in Jerusalem were used.

*Tumours.*—The tumours were 2 transplantable mammary adenocarcinomata. One arose in an outgrowth line of a hyperplastic alveolar mammary nodule appearing in a hormonally hyperstimulated BALB/c female and is designated D7T4S. The other arose spontaneously in a multiparous BALB/cfC3H female infected with the mammary tumour virus (MTV). The reciprocal isogenicity of the 2 sublines BALB/c and BALB/cfC3H was confirmed throughout the course of these experiments by the demonstrated acceptance of second-set skin grafts exchanged between randomly selected female animals from the breeding colony.

*MER.*—The MER preparation was from the same lot described previously (Yron *et al.*, 1975), prepared by Merck, Sharpe and Dohme Research Laboratories (Rahway, New Jersey).

*Therapeutic irradiation.*—A single dose of x-irradiation was given to the tumour site of those animals which were treated with irradiation alone or with irradiation and MER; mice bearing tumour D7T4S received 2000 rad and those with the MTV(+) carcinoma 3000 rad.

*Experimental design.*—In each distinct experiment the mice were distributed at random into groups of 13–17 animals each and were given subcutaneous (s.c.) implants of living tumour tissue (ca. 1 $mm^3$) in the left inguinal area. Treatment was begun 17–21 days after implantation, when 50–70% of the mice in most of the groups had developed growths visible upon inspection of the intact animal; the few groups in which the number of tumour bearing animals at that time was either smaller or greater were excluded from the studies.

Treatment consisted of a single administration of 0·4 mg MER, or of x-rays, or of both administered at 2 different times. Animals of groups not receiving MER at a time when the others received this treatment were given injections of placebo only (0·85% sterile, pyrogen-free saline). MER was administered either by a single s.c. injection of 0·4 mg at a site contralateral to the tumour ( distal [dis] administration ), or 2 simultaneous injections of 0·2 mg each, one directly into the tumour area (" ta ") and one distally s.c.

The mice were observed twice weekly for 95 days after implantation. Most of the animals developed progressively growing cancers at the implantation site regardless of treatment, and many died with large tumour masses during this period. Animals surviving tumour free at the end of the time of observation were included in the calculation; the few mice (less than 5%) which died without visible tumours were excluded from the calculations.

Although none of the treatments succeeded in preventing progressive tumour development, many of the mice given irradiation or MER alone or combined treatment showed a marked slowing of tumour growth and prolonged survival. In these as well as in the previously reported (Yron *et al.*, 1973, 1975) experiments with solid mammary tumours of mice, retardation of tumour development and prolongation of host survival were on the whole closely associated manifestations of limited therapeutic success. The results of therapy in the present study are presented in terms of host longevity after tumour implantation and subsequent treatment, but they accordingly provide as well a parallel indication of effects on tumour growth rate.

*Statistical analysis.*—Differences in the numbers of animals surviving at each time of observation were analyzed by the $\chi^2$ test (two-tailed); comparisons were made between the experimental and control groups of each experiment and between different experimental groups with each other. The results of these comparisons are shown for 3 consecutive periods of measurement, 50–60, 61–70 and 75–90 days after tumour implantation. Where the differences obtained for 2 or more observations within any one such time period were significant, the groups are described as differing significantly for that period; a significant difference at only one time of observation is not considered as representing a significant variation for that entire interval.

### RESULTS

Figure 1 presents the findings obtained in an initial experiment comparing the effects of treatment with MER at a s.c. distal site and both at such a site and in the immediate vicinity of the neoplasms; the agent was injected 21 days after implantation of tumour D7T4S. Mice receiving MER directly into the tumour area as well as elsewhere were afforded no protection. In contrast, treatment with MER by distal injection only,

FIG. 1.—Effect of different routes of MER administration on the survival of mice bearing syngeneic implants of mammary carcinoma D7T4S.

| | Group | Treatment: Days after tumour implantation 17 | 21 |
|---|---|---|---|
| ———————— | 1 | — | MER, 0·4 mg, dis |
| —·—·—· | 2 | — | MER, 0·2mg, dis + 0·2mg, ta |
| ----------- | 3 | Saline, dis | Saline, dis |
| — — — — — | 4 | Saline, dis + ta | Saline, dis + ta |

Statistically significant ($P < 0 \cdot 05$) differences: Group 1 *vs* Group 2, at all 3 intervals; Group 1 *vs* Group 3, at all 3 intervals; Group 1 *vs* Group 4, at 2 intervals.

bestowed significant survival protection, both in comparison with the saline controls and with the animals given MER into the tumour region as well as distally.

The results of a second experiment, with the MTV(+) carcinoma, are depicted in Fig. 2. Here it is seen that the survival time of mice subjected to local plus distal introduction of MER at Day 21 after tumour implantation again did not differ significantly from that of either saline control group. As before, MER administered solely at a place removed from the tumour effected an appreciable prolongation of survival, significantly so when compared with the survival times of mice receiving MER at the tumour site as well as distally, and when compared with the controls given saline at a removed site. However, the protection afforded by distal MER was not significant when compared with the survival pattern of animals injected with saline both into the tumour area and at a removed locus. We have noticed in other studies that traumatic injury sometimes leads to a nonspecific activation of lymphoid cell and/or macrophage cytotoxic capacity, an effect perhaps accruing from the response to injured and necrotic tissues, and it may be that such reactions here reduced the demonstrable protective activity of MER when the comparison was with the group receiving saline directly into the tumour location.

The observations made in a third experiment conducted simultaneously with the second are shown in Fig. 3. Mice given MER both distally and at the place of transplants of the MTV(+) tumour

FIG. 2.—Effect of different routes of MER administration on the survival of mice bearing syngeneic implants of an MTV(+) mammary carcinoma.

| | Group | Treatment Days after tumour implantation 21 | 25 |
|---|---|---|---|
| ———————— | 1 | Mer, 0·4mg, dis | — |
| —·—·—·— | 2 | MER, 0·2mg, dis + 0·2mg, ta | — |
| ---------------- | 3 | Saline, dis | Saline, dis |
| — — — — — | 4 | Saline, dis + ta | Saline, dis + ta |

Statistically significant differences: Group 1 *vs* Group 2, at 1 time interval; Group 1 *vs* Group 3, at 1 time interval.

succumbed significantly sooner than either of the control groups. On the other hand, the animals treated by distal administration only, survived significantly longer than either those receiving some of the MER into the tumour area or those receiving saline at a distal s.c. site. The distally treated MER animals also showed some survival protection compared with mice given saline into the tumour site and elsewhere, but here too, this effect was not statistically significant.

In a further experiment, the effects of these different routes of MER introduction were compared in animals receiving additional therapy by means of x-rays. It is seen from Fig. 4 that mice given x-rays on Day 17 and MER s.c. on Day 21 after implantation of tumour D7T4S died more slowly with progressively growing cancers than the animals in any of the other groups. The differences in survival times between these mice and those of both control groups and of the group given x-rays alone were significant. Radiation therapy by itself improved survival significantly at one time interval over that of animals given saline at a distal s.c. site but not over that of animals receiving saline into the tumour focus as well. The combined therapeutic intervention of x-rays plus MER administered both to the tumour site and distally effected significant survival protection compared with both the control groups, but did not improve the results obtained with irradiation alone. Thus in this experiment, combined therapy proved superior to x-rays alone only where treatment with MER was to a distal location and not into the tumour region.

In a parallel experiment with the same carcinoma in which treatment with MER (on Day 17) preceded therapeutic irradia-

FIG. 3.—Effect of different routes of MER administration on the survival of mice bearing syngeneic implants of an MTV(+) mammary carcinoma.

| | | Treatment Days after tumour implantation | |
|---|---|---|---|
| | Group | 21 | 25 |
| ———————— | 1 | — | MER, 0·4mg, dis |
| —·—·—·—· | 2 | — | MER, 0·2mg, dis + 0·2mg, ta |
| ----------- | 3 | Saline, dis | Saline, dis |
| — — — — — | 4 | Saline, dis + ta | Saline, dis + ta |

Statistically significant differences: Group 1 *vs* Group 2, at all 3 intervals; Group 1 *vs* Group 3, at 1 interval; Group 2 *vs* Group 3, at 2 intervals; Group 2 *vs* Group 4, at all 3 intervals.

tion (Day 21), x-rays alone exerted significant survival prolongation effects, as did combined irradiation–MER treatment, regardless of the route of MER administration. In this one experiment, however, injection of MER both into the tumour area and distally was slightly, but significantly, more efficacious than introduction of the agent at a removed s.c. locality only. Moreover, whereas there was no significant difference in the degree of protection achieved by irradiation alone and by combined treatment in which MER was applied both distally and in the tumour area, x-ray therapy alone was significantly better than joint intervention with MER injected only distally.

### DISCUSSION

The tumour therapy experiments here described were conducted with 2 mammary carcinomata of BALB/c mice, both of which develop rapidly and fatally in the syngeneic hosts. Neither MER nor therapeutic irradiation, nor combined treatment, succeeded in effecting cures. However, these modalities of intervention often slowed the growth of the neoplasms and prolonged the survival of the animals commensurately.

Treatment with MER alone bestowed significant survival protection in all of 3 experiments when the material was administered at a subcutaneous site distal from the tumour focus. When, however, injection of MER was both at such a distal site and directly into the tumour location, no protection was afforded and significantly shortened survival was seen in one instance.

In 2 additional experiments in which tumour bearing mice were treated with both MER and focal x-irradiation, therapy

FIG. 4.—Effect of x-irradiation and MER administration by different routes on the survival of mice bearing syngeneic implants of mammary carcinoma D7T4S.

| | | Treatment: Days after tumour implantation 17 | 21 |
|---|---|---|---|
| | Group | 17 | 21 |
| ———————— | 1 | X-rays | MER, 0·4mg, dis |
| · · · · · · · · · · | 2 | X-rays | Saline, dis |
| —·—·—·— | 3 | X-rays | MER, 0·2mg, dis + 0·2mg, ta |
| ------------ | 4 | Saline, dis | Saline, dis |
| — — — — — | 5 | Saline, dis + ta | Saline, dis + ta |

Statistically significant differences: Group 1 *vs* Group 2, at 2 intervals; Group 1 *vs* Group 4, at all 3 intervals; Group 1 *vs* Group 5, at 2 intervals; Group 2 *vs* Group 4, at 1 interval; Group 3 *vs* Group 4, at all 3 intervals; Group 3 *vs* Group 5, at 2 intervals.

by irradiation alone as well as combined therapy proved effective in survival prolongation. Where combined treatment was used, significant efficacy was demonstrated when MER administration was only at a distal site, and also when it was both into the tumour locality and distally. In one of these experiments, joint treatment with x-rays and MER proved superior to irradiation alone only when MER administration was at a site distal to the tumour, and not when the fraction was introduced both into the tumour region and away from it. In the second experiment, in which the sequence of x-ray and MER treatment was reversed, joint therapy was in neither instance more effective than x-rays by themselves, and was less beneficial than irradiation alone when the MER component of the combined modality was given at a distal site.

These observations thus indicate that in most instances MER applied solely at a s.c. location contralateral to the tumour implant is more likely to bestow a degree of protection against the rapidly fatal progression of 2 solid mammary carcinomata than introduction of the substance at the tumour site as well as distally. Although these findings do not permit generalization as to the advantage of introducing a nonspecific modulator of immunological responsiveness and antitumour resistance at a place removed from a neoplastic focus, they are consistent with the observations of many other investigators that nonspecific immunotherapy of neoplastic disease is not necessarily contingent on injection of active agents into a tumour mass (Borsos and Rapp, 1973; Schmidtke and Simmons, 1974). The findings certainly cast doubt on any attempt to generalize to the opposite effect—that local administration of nonspecific immunostimulators is necessary—from individual test models in which such route of introduction has seemed superior (Rapp, 1973).

It also appears from the results described here that injection of placebo (isotonic saline) into or adjacent to a tumour can elicit effects sufficient to obscure the therapeutic action of MER and even of x-irradiation in parallel experimental groups. Such consequences of treatment must be taken into account in the design of all tumour immunotherapy studies, and they may be indicative of one of the mechanisms of nonspecifically induced immunological intervention. Damage to tissues, necrosis and inflammatory changes no matter how induced can be envisaged to impinge on host immunological reactivity in a variety of ways, and directly on tumour cells in the vicinity as "innocent bystanders" of the event. It would appear probable that both specific and truly nonspecific reactions come into play in the stimulation of immune responses against neoplastic cells and a seemingly nonspecific immunostimulator may be capable of eliciting a variety of effects of immunological consequence, especially when it is a complex natural product. It would be dangerous to conclude that the parameters governing the behaviour of any one such agent against a given tumour represent the conditions demanded for efficacy by all similar agents against that tumour, or by the same substance against other neoplasms.

The authors express their thanks to Miss Yafa Bot and Mrs Rama Siman-Tov for excellent technical assistance.

Supported by Research Contract No. NIH 70-2208 from the National Cancer Institute, National Institutes of Health; The Lautenberg Endowment, Concern Foundation Inc and Mr and Mrs Laurence A. Tisch.

### REFERENCES

Ben-Efraim, S., Constantini-Sourojon, M. & Weiss, D. W. (1973) Potentiation and Modulation of the Immune Response of Guinea-pigs to Poorly Immunogenic Protein-hapten Conjugates by Pretreatment with the MER Fraction of Attenuated Tubercle Bacilli. *Cell. Immunol.*, **7**, 370.

Ben-Efraim, S., Teitelbaum, R., Ophir, R., Kleinman, R. & Weiss, D. W. (1974) Non-specific Modulation of Immunological Responsiveness in Guinea-pigs and Mice by the MER Mycobacterial Fraction: Influence of Conditions of MER Treatment and Specific Immunization, and Effect of MER on Early Stages in the Immune Response. In *Immunological Parameters of Host–Tumor Relationships*, Vol. III. Ed. D. W. Weiss. New York: Academic Press. p. 158.

Borsos, T. & Rapp, H. J. (1973) Editors, Conference on the Use of BCG in Therapy of Cancer. *Natn. Cancer Inst. Monog.*, **39**.

Gery, I., Baer, A., Stupp, Y. & Weiss, D. W. (1974) Further Studies on the Effects of the Methanol Extraction Residue Fraction of Tubercle Bacilli on Lymphoid Cells and Macrophages. In *Immunological Parameters of Host–Tumor Relationships*, Vol. III. Ed. D. W. Weiss. New York: Academic Press. p. 170.

Haran-Ghera, N. & Weiss, D. W. (1973) Effect of Treatment of C57Bl/6 Mice with the MER Fraction of BCG on Leukemogenesis by the Radiation Leukaemia Virus (RLV). *J. natn. Cancer Inst.*, **50**, 229.

Izak, G., Manny, N., Weiss, D. W. & Stupp, Y. (1974) Immunotherapy in Acute Myelocytic Leukemia. *Proc. Int. Symp. Standardization in Hematology and in Clinical Pathology.* Ospedale "Casa Sollaievo De La Sofferenza" San Giovanni Foggia, 12–15 September.

Kuperman, O., Feigis, M. & Weiss, D. W. (1973) Reversal by the MER Tubercle Bacillus Fraction of the Suppressive Effects of Heterologous Antilymphocytic Serum (ALS) on the Allograft Reactivity of Mice. *Cell. Immunol.*, **8**, 484.

Moertel, C. G., Ritts, R. E., Schutt, A. J. & Hahn, R. G. (1975) A Phase 1 Study of Methanol Extraction Residue of BCG (MER-BCG). *Proc. Am. Ass. Cancer Res.*, **16**, 143.

Rapp, H. J. (1973) A Guinea-pig Model for Tumor Immunology. A Summary. In *Immunological Parameters of Host–Tumor Relationships*, Vol. II. Ed. D. W. Weiss. New York: Academic Press. p. 162.

Richman, S. P. (1975) Phase 1 Study of Immunotherapy with Methanol Extraction Residue of BCG (MER). *Proc. Am. Soc. clin. Oncol.*, **16**, 227.

Schmidtke, J. R. & Simmons, R. L. (1974) Experimental Models of Tumor Immunotherapy. In *Clinical Immunobiology*, Vol. 2. Ed. F. H. Bach and R. A. Good. London: Academic Press. p. 265.

WEISS, D. W. (1972) Nonspecific Stimulation and Modulation of the Immune Response and of States of Resistance by the MER Fraction of Tubercle Bacilli. *Natn. Cancer Inst. Monog.*, **35**, 157.

WEISS, D. W., STUPP, Y., MANNY, N. & IZAK, G. (1975) Treatment of Acute Myelocytic Leukemia (AML) patients with the MER Tubercle Bacillus Fraction. A Preliminary Report. *Transplantn Proc*, VII, No. 1, Suppl. I. p. 545.

WEISS, D. W. & YASHPHE, D. J. (1973) Nonspecific Stimulation of Antimicrobial and Antitumor Resistance and of Immunological Responsiveness by the MER Fraction of Tubercle Bacilli. In *Dynamic Aspects of Host–Parasite Relationships*, Vol. 1. Ed. A. Zuckerman and D. W. Weiss. New York: Academic Press. p. 163.

YRON, I., COHEN, D., ROBINSON, E., HABER, M. & WEISS, D. W. (1975) Effects of MER and Therapeutic Irradiation against Established Isografts and Simulated Local Recurrence of Mammary Carcinomas. *Cancer Res.*, **35**, 1779.

YRON, I., WEISS, D. W., ROBINSON, E., COHEN, D., ADELBERG, M. G., MEKORI, T. & HABER, M. (1973) Immunotherapeutic Studies in Mice with the Methanol Extraction Residue (MER) Fraction of BCG: Solid Tumors. *Natn. Cancer Inst. Monog.*, **39**, 33.